# News and Miscellany

**Published:** 1900-10

**Authors:** 


### News and Miscellany.

**New subscribers** who send us $1.00 now, will receive October, November and December numbers free.

**Dr. F. S. White**, of Terrell, Texas, is at the New York Polyclinic. Dr. White will send the JOURNAL some "newsy notes" for next issue.

**The "Red-Back"** sends greeting to its subscribers, and congratulates them on the present high price of cotton! This insures them good collections, and we ask to be remembered.

Druggist: "Pills, my dear?"
Little Girl: "Yes, please, sir."
Druggist· "Anti-bilious?"
Little Girl: "No, Uncle is."

**Send a Postal Card** to Battle & Co., St. Louis, for their set of pamphlets illustrating, with colored engravings, the fractures of the long bones, showing the regional anatomy. Free if the "Redback" be mentioned.

**The American Journal of Surgery and Gynecology** (Lanphear), St. Louis, $1.00 per year. The TEXAS MEDICAL JOURNAL, $1.00 per year. By special arrangement we will send both journals one year for $1, if cash accompanies the order.

**Dr. R. A. Goethe,** of Boerne, Texas, has just returned from a six months' course of special laboratory and bedside study in New York, and will at once establish, at Boerne, a pathological laboratory and hopes to be of assistance to his medical friends, in difficult diagnosis.

**Death of Dr. Doak.**—Dr. A. V. Doak, of Taylor, Texas, died at his home in that place on September 14th (ult.). Dr. Doak was one of the pioneers of Taylor, and did more towards building up and developing that city than almost any other one person. He made and spent several fortunes there in investments and speculations. He was a well known and popular physician.

**Death of Dr. Hunter McGuire.**—Dr. McGuire suffered a paralytic stroke some six months ago from which he never recovered. He died at his home in Richmond, on 19th of Septemtember, ult.

---

**The Mississippi Medical Record** is offering to its subscribers a Clarke and Roberts No. 80, $100.00 surgical table for the best original essay on any medical or surgical subject. Contest closes April 1, 1901. For particulars write to the Mississippi Medical Record, Vicksburg, Miss.

---

**For Sale:**—$1600 practice, nice home and office, in country village, Central Texas, No opposition, people all Americans. Terms, $500, $300 cash, balance to suit. If you mean business write for particulars. Address Dr. L. C. B———

Care TEXAS MEDICAL JOURNAL.

---

**New Orleans Polyclinic.**—Physicians will find the Polyclinic an excellent means for posting themselves upon modern progress in all branches of medicine and surgery. The specialties are fully taught, particularly laboratory work. Fourteenth annual session opens November 12, 1900. For further information address Dr. Isadore Dyer, Secretary, New Orleans Polyclinic, New Orleans.

---

**The Texas Medical College** (Medical Department University of Texas) Galveston, announces (see page advertisement) that owing to the disaster to Galveston, the annual session has been posponed to November 15, prox. Examinations for matriculation and advanced standing will be held the week preceding the opening of the lecture session. It is gratifying to state that the splendid college buildings were but little damaged.

---

**Dr. I. N. Love,** the owner and editor of *Love's Medical Mirror*, St. Louis, has removed to New York. His address is "The Iroquois," 49, W. 44th St. The *Mirror* will still be issued from St. Louis, but the editorial policy and management will be dictated and directed by Dr. Love, in New York. The *Mirror* will be represented in St. Louis by Drs. Boylen, Lee, and Hall. Dr. Hall has been Dr. Love's assistant on the *Mirror* several years. We learn that Dr. Love is to be connected with one of the post graduate medical schools in New York.

**Dr. August H. Schenk,** of Kenney, Texas, died very suddenly, of heart failure, in his office in that place, August 24th, last. Dr. Schenk was in his 34th year of age. He was recognized as one of the ablest and most progressive physicians of his section. He was a native Texan; graduated in medicine from the Medical Department of the University of Louisville, Kentucky, in 1888; delivered the valedictory for his class. He then went to Bellvue Hospital Medical College one term. Located in Kenney and practiced medicine there to the date of his death. In 1891 Dr. Schenk was married to Miss Fannie Kennedy, of Brenham. She and two sons survive him, as well as his mother and three sisters.

---

**Death of Dr. Scott.**—Dr. Preston B. Scott died at his home in Louisville, September 24 (ult.). In our August number we published a portrait of this distinguished Confederate Surgeon, together with a brief sketch of his life. At the great Confederate reunion at Louisville, June 1–4, 1900, an association of ex-Confederate army and navy surgeons was organized and Dr. Scott was elected president; Dr. I. M. Keller, of Hot Springs, was elected vice-president, and will succeed, of course, to the presidency. A large part of the success of the reunion was due to Dr. Scott's indefatigable labors, and at the close of the meeting, being very much fatigued, he went to the mountains of Virginia to rest nd recuperate. We learn that on his return to Louisville he developed typhoid fever from which he died. Dr. Scott was one of the illustrious medical men of the war-time, and was universally loved, admired and esteemed by the people of the South for his pure life and noble qualities, as well as for his services to the Lost Cause.

---

**New Home for J. B. Lippincott Company.**—An important transaction has just been concluded by which a number of old-fashioned dwelling houses on East Washington Square have passed from the ownership to the heirs of the famous lawyer, Horace Binny, and will soon be torn down to make way for a fine building to be occupied by J. B. Lippincott Company, whose old home on Filbert Street, above Seventh, was burned down some months ago. Possession is to be gived by September 14, and it is expected that the demolition of the old structures will begin soon after. The site is considered a very eligible one for the Lippincott Company, as it has light on three sides, is very central and they wi'l be enabled to promptly issue and increase their excellent line of medical publications by standard authorities.

By the way, their new catalogue, just issued, is handsomely illustrated with excellent portraits of many of America's leading medical writers.

Many historic recollections cluster about the properties just sold. They stand on the ground once occupied by the old Walnut Street prison, built before the Revolution, and in which during the struggle the English confined American prisoners during the former's occupation of Philadelphia.

---

**A New Medical College in Texas.**—"The University of Dallas" is the name of a new medical college to be inaugurated at Dallas, Texas. A charter has been obtained by an association of physicians—and a board of trustees appointed, of which Dr. S. H. Stout, the Medical Director of all the hospitals in the Southern Confederacy during the war, is President. The charter authorizes the incorporators to teach law, medicine, the arts and sciences, and to confer all professional and academic degrees usually granted by universities. At present only the School of Medicine will be inaugurated. The faculty elected by the trustees at their first meeting, recently, consists of the following well-known physicians: Dr. J. E. Gilcreest, Gainesville; Dr. V. B. Armstrong, Dallas; Dr. B. E. Hadra, (President Texas Medical Association) Waco; Dr. S. E. Milliken, Dallas; Dr. Joe Becton, Greenville; Dr. L. Ashton, Dallas; Dr. C. M. Rosser, Dallas; Dr. A. F. Beddo, Dallas and Dr. J. B. Titterington, of Dallas, Dean of the Faculty.

It is the purpose to begin lectures next month. We are not informed as to further details. They can be had by writing to the Dean, Dr. Titterington.

---

### Summer Disorders of Children.

---

Each year, as the heated term approaches, the physician feels with dread that he cannot avoid being frequently called upon to treat the so-called "summer disorders" of infants.

It is a very difficult task to cope with diseases of infants, and especially is this true of the ills common among them during the summer months.

If proper attention is given to the feeding of infants there is no reason why they should suffer more in summer than at any other season of the year, but "proper attention" in this instance means giving an infant the proper food.

The best food for an infant is his mother's milk, and the best substitute that can be given him for his mother's milk is some other infant's mother's milk, and if this cannot be obtained cow's milk may be successfully used if intelligently prepared.

Unfortunately, it is very difficult to keep cow's milk "fresh" during hot weather. An infant must be fed at short intervals, and milk from the cow can only be obtained at long intervals, consequently the danger of giving him "sour" milk is very great.

It is for this reason that many experienced infant care-takers prefer artificial infant foods to cow's milk.

As is well known, most bottle-fed infants are overfed, and it is to this overfeeding that so many of the intestinal and gastric disorders of infants are due.

If the physician will instruct mothers to feed their infants at regular stated intervals, and underfeed rather than overfeed, many of the disorders that occur among them during the hot weather can be avoided.

An abundance of fresh air should be given to the infant during the summer months, and it is far better to have them suffer from hunger than from overfeeding.—*Gaillard's Medical Journal.*

---

### The Injurious Effects of Improperly Constructed School Chairs.

---

J. S. Stone says that lateral curvature of the spine is a disease originating almost always during school life, and is much more frequent among girls than among boys. The ordinary type of lateral curvature is that of the writing position. In Nuremburg twice as many incorrect postures were found among children writing the oblique as among those writing the vertical script; in Munich two and a half times as many; in Fürth and Würzburg four times as many. The chair back has always been the great problem. There are two essentials to a proper back. In the first place, the shoulders should never be forced forward, and in the second the physiological anterior lumbar curve should be maintained. It has been found a matter of commercial value to provide the typewriter with an adjustable automatic spring back constructed on hygienic principles. Are not the health and well-being of the growing children of the community of as great consequence as the comfort of the typewriters, in whom, because of greater age, deformities are much less likely to occur? No attitude, no matter how good in itself, can be long maintained without fatigue. Professor Mille has devised a chair to allow a change of distance, to allow an upright and a reclining position, and to allow for some changes in the antero-posterior curves of the spine, while at the same time always giving a firm lumbar support.—*Boston Medical and Surgical Journal.*

